# Sucroferric oxyhydroxide for hyperphosphatemia: a review of real-world evidence

**DOI:** 10.1007/s40620-021-01241-5

**Published:** 2022-02-09

**Authors:** Daniel W. Coyne, Stuart M. Sprague, Marc Vervloet, Rosa Ramos, Kamyar Kalantar-Zadeh

**Affiliations:** 1 Division of Nephrology, Washington University School of Medicine, 660 S. Euclid Ave., CB 8129, St. Louis, MO 63110 USA; 2 Division of Nephrology and Hypertension, NorthShore University Health System, University of Chicago Pritzker School of Medicine, Evanston, IL USA; 3 Department of Nephrology and Amsterdam Cardiovascular Sciences (ACS), Amsterdam University Medical Center, Amsterdam, The Netherlands; 4 NephroCare Spain, Nephrology, Madrid, Spain; 5 Irvine School of Medicine, University of California, Orange, CA USA

**Keywords:** Chronic kidney disease, Hemodialysis, Peritoneal dialysis, Phosphate binder, Phosphorus

## Abstract

Hyperphosphatemia is a common complication in dialysis-dependent patients with chronic kidney disease. Most dialysis-dependent patients need oral phosphate binder therapy to control serum phosphorus concentrations. Most phosphate binders have a high daily pill burden, which may reduce treatment adherence and impair phosphorus control. Sucroferric oxyhydroxide is a potent iron-based phosphate binder approved for use in dialysis-dependent patients in 2013. A randomized controlled trial of sucroferric oxyhydroxide demonstrated its efficacy for reduction of serum phosphorus with a lower pill burden than sevelamer carbonate. Clinical trials carefully select patients, monitor adherence, and routinely titrate medications to a protocol-defined goal. Consequently, trials may not reflect real-world use of medications. Since its approval, we and others have performed retrospective and prospective analyses of sucroferric oxyhydroxide in real-world clinical practice in > 6400 hemodialysis and approximately 500 peritoneal dialysis patients in the USA and Europe. Consistent with the clinical trial data, real-world observational studies have demonstrated that sucroferric oxyhydroxide can effectively reduce serum phosphorus with a lower daily pill burden than most other phosphate binders. These studies have also shown sucroferric oxyhydroxide provides effective serum phosphorus control in different treatment settings, including as monotherapy in phosphate binder-naïve patients, in patients switching from other phosphate binders, or when used in combination with other phosphate binders. These observational studies indicate a favorable safety and tolerability profile, and minimal, if any, systemic iron absorption. This article reviews the key results from these observational studies of sucroferric oxyhydroxide and evaluates its role in the management of hyperphosphatemia in clinical practice.

## Introduction

Hyperphosphatemia is a frequent complication in patients with dialysis-dependent chronic kidney disease [1]. Elevated serum phosphorus is a major factor in the development of secondary hyperparathyroidism, and is associated with vascular calcification, increased cardiovascular events, and higher mortality in dialysis patients [2–7]. Experimental studies strongly suggest a causal role for hyperphosphatemia for these clinical events [8].

While randomized controlled trials are lacking, large observational cohort studies of dialysis patients have shown that reduction of elevated serum phosphorus is associated with improved survival [9, 10]. Guideline organizations provide different targets for phosphorus control in dialysis patients. The National Kidney Foundation’s Kidney Disease Outcomes Quality Initiative guidelines [11] recommend targeting a serum phosphorus of 3.5–5.5 mg/dl, while the Kidney Disease Improving Global Outcomes guidelines [12] recommend lowering phosphorus toward “normal” (< 4.6 mg/dl).

The majority of dialysis patients need treatment with oral phosphate binders (PBs) to reduce gastrointestinal (GI) absorption of phosphate and achieve serum phosphorus control [13]. Data from cross-sectional studies show that dialysis patients treated with PB therapy have significantly lower mortality rates, compared with those who do not receive PBs, even after propensity score matching and adjustment for nutritional status [14–16]. Nevertheless, more than a third of dialysis patients in the USA and Europe have serum phosphorus concentrations above 5.5 mg/dl [17, 18].

Several PBs are currently available. Most PBs have a high daily pill burden [19, 20], which may lead to poor adherence. Poor PB adherence associates with higher serum phosphorus [21, 22]. An analysis of global data showed that 45% of dialysis patients skipped taking their PB at least once in the previous month, and 57% of US patients reported doing so [22]. Key attributes of an optimal PB therapy include a high phosphate-binding capacity across the pH range in the GI tract, a low daily pill burden, minimal systemic absorption, a good safety profile, high tolerability, and low cost [23].

Sucroferric oxyhydroxide (SO; Velphoro^®^) is a potent, iron-based PB with a low daily pill burden, approved for the control of serum phosphorus concentrations in patients with chronic kidney disease undergoing dialysis [24, 25]. SO is approved in the USA (2013), Europe (2014), and several other countries. The product is composed from a mixture of sucrose, starches, and the active moiety, polynuclear iron(III)-oxyhydroxide. It is formulated as chewable, berry-flavored tablets, which each contain 500 mg of iron. The recommended starting dose of SO is 3 tablets (1500 mg) per day, taken as 1 tablet (500 mg) 3 times daily with meals. The dose of SO should be titrated in increments of 1 tablet (500 mg) each day every 2–4 weeks until the target serum phosphorus concentration is reached. The maximum daily dose of SO evaluated in clinical studies was 6 tablets per day (3000 mg) [25]. The SO 24-week Phase 3 clinical trial, conducted in 1,055 hemodialysis and peritoneal dialysis patients, demonstrated that SO was non-inferior to sevelamer carbonate (“sevelamer”) for reduction of serum phosphorus after 12 weeks, with a lower daily pill burden (2.8 pills/day vs 7.6 pills/day) and better treatment adherence (82.6% vs 77.2% at Week 24) [26]. These reductions in serum phosphorus with SO were also sustained during the subsequent 28-week extension study [27]. During the Phase 3 trial and its extension study, the most commonly reported adverse events with SO were mild transient diarrhea and discolored feces [26, 27].

Subgroup analyses of the Phase 3 data showed a similar long-term efficacy and safety profile for SO in patients who were undergoing peritoneal dialysis [28] and in African American patients [29].

A post hoc analysis of iron-related parameters showed a small but statistically significant increase in transferrin saturation (TSAT) with SO, compared with sevelamer, at 24 weeks (+ 4.6% vs + 0.6%, *p* = 0.003), while the increase of mean serum ferritin did not differ significantly between SO and sevelamer (+ 119 ng/ml vs + 56 ng/ml, respectively, *p* = not significant) [30]. The small increase in TSAT with SO use suggests a low level of iron absorption. Consistent with this finding, an Fe-59 radiolabeled-SO iron absorption study in eight hemodialysis patients reported median uptake of 0.02% of elemental iron present in SO (range 0–0.04%) [31]. In the Phase 3 trial, use of intravenous (IV) iron was common in both study arms (> 70%) and was likely the major driver for most changes in iron-related parameters [30]. Although the use of IV iron and erythropoiesis-stimulating agents (ESAs) was similar at baseline, IV iron use was slightly lower in the SO arm than in the sevelamer arm over Weeks 1 to 24 (69.3% vs 75.8%, respectively) and during Weeks 24 to 52 (63.0% vs 68.3%, respectively). Concomitant ESA therapy was also lower in the SO arm vs the sevelamer arm during Weeks 1–24 (83.2% vs 86.8%) and Weeks 24 to 52 (80.8% vs 88.1%; *p* = 0.0252) [30].

A post hoc analysis of changes in mineral bone disorder markers during the Phase 3 trial data pooled the groups because SO and sevelamer had similar effects [32]. After 1 year of treatment, median intact fibroblast growth factor-23 (FGF-23) decreased by 64% (*p* < 0.001 vs baseline). Serum calcium concentrations remained unchanged during this period. The bone formation markers, bone-specific alkaline phosphatase, and osteocalcin increased, which may indicate a benefit of SO and sevelamer therapy on bone metabolism [32].

Prospective clinical trials of SO were conducted in specialist centers, enrolled select dialysis patients, and continually monitored drug adherence and titrated medications to target phosphorus. These studies differ from the real-world setting, limiting external validity of clinical trial findings. To assess aspects of routine clinical use of SO, a series of observational studies were performed in the USA and Europe since 2013 (summarized in Table 1). The key results from these observational studies of SO are presented below.

**Table 1:** Overview of key real-world studies evaluating sucroferric oxyhydroxide in dialysis patients

Author	Location of study population	Study design and SO follow-up duration	Patient population analyzed	Summary of key results
**FKC database studies**				
Coyne et al. (2017) [33]	USA	Retrospective cohort study SO follow-up: up to 6 months	•1,029 adult in-center HD patients switched to SO monotherapy from another PB and received up to 6 months of SO therapy •2 × patient cohorts analyzed: –1–3 months of SO prescription (SO 1–3; *n* = 1,029) –4–6 months of SO prescription (SO 4–6; *n* = 424)	% patients with sP ≤ 5.5 mg/dl (BL vs Months 3 and 6) •SO 1–3: 13.9% vs 26.1% at Month 3 (*p* < 0.001) •SO 4–6: 15.6% vs 30.4% at Month 6 (*p* < 0.0001) PB pill burden (BL vs Months 3 and 6) •SO 1–3: 9.6 vs 3.8 pills/day (*p* < 0.001) •SO 4–6: 9.7 vs 4.0 pills/day (*p* < 0.0001)
Kendrick et al. (2019) [34]	USA	Retrospective cohort study SO follow-up: 12 months	•530 adult, in-center HD patients switched to SO monotherapy from another PB. All patients were required to have 12 months of uninterrupted SO prescriptions •Subgroup analyses were also performed for African American (*n* = 217) and Hispanic (*n* = 87) patients	% patients with sP ≤ 5.5 mg/dl (BL vs Q1 − Q4) •Overall cohort: 17.7% vs 24.5 − 36.4% (*p* < 0.0001) •African Americans: 14.3% vs 23.0 − 34.1% (*p* = 0.004) •Hispanics: 18.4% vs 28.7 − 39.1% (*p* < 0.02 for Q1, Q3, Q4) PB pill burden (BL vs Q1 − Q4) •Overall cohort: 8.5 vs 4.3 pills/day (*p* < 0.0001) •African Americans: 8.9 vs 4.1–4.5 pills/day (*p* < 0.0001) •Hispanics: 8.9 vs 4.1–4.4 pills/day (*p* < 0.0001)
Coyne et al. (2020) [35]	USA	Retrospective comparative cohort study SO follow-up: 24 months	•Patients who received 2 years of uninterrupted SO therapy (mSO, *n* = 222) were compared with patients who discontinued SO therapy (dSO, *n* = 596) within 90 days of first prescription and switched to other PBs	% patients with sP ≤ 5.5 mg/dl (BL vs Q1 and Q8) •mSO: 20.7% vs 36.9% and 45.0% (*p* < 0.001) •dSO: 16.1% vs 29.0% and 31.9% (*p* < 0.001) PB pill burden (BL vs Q8) •mSO: 7.5 vs 4.4 pills/day (*p* < 0.001) •dSO: 9.1 vs 9.3 pills/day (*p* = 0.3) All-cause hospitalizations •mSO patients had 35.6 fewer hospitalizations per 100 patient-years (incidence rate ratio, 0.75 [95% CI, 0.58–0.96])
Kalantar-Zadeh et al. (2018) [36]	USA	Retrospective cohort study SO follow-up: 12 months	•172 adult in-center PB-naïve HD patients who initiated SO as a first-line PB therapy •A subgroup analysis was performed on 44 patients who were new to dialysis treatment (i.e. within their first year of dialysis)	% patients with sP ≤ 5.5 mg/dl (BL vs Q1 − Q4) •Overall cohort (n = 172): 23.7% vs 32.6 − 38.8% (*p* < 0.0001) •New to dialysis (n = 44): 31.8% vs 40.9 − 52.4% (*p* < 0.0001) SO pill burden (Q1 − Q4) •Overall cohort (n = 172): 3.9 − 4.1 pills/day (*p* < 0.0001) •New to dialysis (n = 44): 3.7 − 3.9 pills/day (*p* < 0.0001)
Kalantar-Zadeh et al. (2019) [37]	USA	Retrospective cohort study SO follow-up: 24 months	•59 adult in-center PB-naïve HD patients who initiated SO as a first-line PB therapy within their first year of dialysis treatment	% patients with sP ≤ 5.5 mg/dl (BL vs Q1 − Q8) •37% vs 42 − 49% (*p* < 0.001) SO pill burden (Q1 − Q8) •4.4 − 5.1 pills/day
Molony et al. (2020) [38]	USA	Retrospective cohort study SO follow-up: up to 12 months	•234 adult in-center HD patients prescribed SO in combination with other PB(s) for at least 120 days	% patients with sP ≤ 5.5 mg/dl (BL vs Q1 − Q4) •19.0% vs 33–40% (*p* < 0.001) PB pill burden (BL vs Q1 − Q4) •12.3 vs 15.8 − 12.3 pills/day (*p* < 001 at Q1; *p* = 0.9 at Q4)
Kalantar-Zadeh et al. (2018) [40]	USA	Retrospective cohort study SO follow-up: up to 6 months	•258 adult peritoneal dialysis patients prescribed SO monotherapy for up to 6 months	% patients with sP ≤ 5.5 mg/dl (BL vs Month 6) •26.0% vs 44.4% (*p* < 0.001) PB pill burden (BL vs Month 6) •10.0 vs 4.3 pills/day (*p* < 0.0001)
DaVita Inc. database				
Gray et al. (2019) [41]	USA	Retrospective cohort study SO follow-up: up to 6 months	•490 adult in-center HD patients converted to SO monotherapy from another PB therapy •A subgroup analysis was performed on 30 patients not using the DaVita pharmacy’s automated refill-management services to evaluate changes in PB medical possession ratio following the switch to SO	% patients with sP ≤ 5.5 mg/dl (BL vs follow-up) •22.0% vs 30,0% (*p* < 0.001) PB pill burden (BL vs follow-up period) •10.8 vs 5.5 pills/day (*p* < 0.001) Mean PB medication possession ratio (BL vs Month 6) •0.68 vs 0.80 (*p* = 0.01)
**FMC EuCliD**^®^ **database**				
Ramos et al. (2020) [42]	Europe	Retrospective cohort study SO follow-up: up to 12 months	•1,096 HD patients from five European countries (France, Italy, Portugal, Russia, Spain) newly prescribed SO for up to 1 year as part of routine clinical practice •Subgroup analysis was performed on the following patient subgroups –188 PB-naïve patients treated with SO monotherapy (SO subgroup) –53 PB-pretreated patients switched to SO monotherapy (PB → SO subgroup) –796 PB-pretreated patients receiving SO in addition to another PB (PB + SO subgroup)	% of patients with sP ≤ 5.5 mg/dl (BL vs Q1–Q4) •Overall cohort: 41.3% vs 56.2 − 62.7% (*p* < 0.0001) •SO subgroup: 49.5% vs 62.5–75.2% (*p* < 0.0001 for Q1, Q2, Q3) •PB → SO subgroup: 58.5% vs 53.6–68.0 (n.s.) •PB + SO subgroup: 38.1% vs 53.9–60.9% (*p* < 0.0001 Q1–Q4); PB pill burden (BL vs Q1–Q4) •Overall: 6.3 vs 5.0 − 5.3 pills/day •SO subgroup: 0 vs 2.1 − 2.3 pills/day •PB → SO subgroup: 2.1 vs 2.6 − 2.8 pills/day •PB + SO subgroup: 6.5 vs 6.0 − 6.2 pills/day
**VERIFIE post-authorization study**				
Vervloet et al. (2020) [43]	Europe	Non-interventional, prospective, post-authorization safety study SO follow-up: up to 36 months	•1,402 dialysis patients from seven European countries (France, Germany, Greece, Italy, Portugal, Spain, and United Kingdom) prescribed SO as part of routine clinical practice •1,365 patients were eligible for safety analysis and 1,322 patients were evaluable for SO effectiveness analysis	Safety results •531/1,365 patients (39%) had ≥ 1 ADR during SO treatment •Most frequent ADRs: diarrhea (14% patients) and discolored feces (9% patients), mainly mild/moderate in severity •Fatal events occurred in 119 patients (8.7%); none were considered treatment-related •Small increases in mean serum ferritin and TSAT: -Ferritin: 377 µg/l at BL up to 444 µg/L at Month 24 (Δ BL: + 75 µg/l; *p* < 0.05) -TSAT: 26.1% at BL up to 29.0% at Month 3 (Δ BL: + 2.1%; *p* < 0.001) Effectiveness results % of patients with sP ≤ 5.5 mg/dl (BL vs follow-up period) •30% vs 47 − 63% (*p* < 0.001) SO pill burden •Overall cohort: 2.3 SO pills/day •SO monotherapy subgroup: 2.5 SO pills/day •SO combination subgroup: 2.3 SO pills/day

*ADR*, adverse drug reaction; *BL*, baseline; *FKC*, Fresenius Kidney Care; *HD*, hemodialysis; *n.s*., not statistically significant; *PB*, phosphate binder; *SO*, sucroferric oxyhydroxide

### Real-world effectiveness of sucroferric oxyhydroxide

Several effectiveness studies have utilized medical databases of large US and European dialysis organizations to retrospectively analyze de-identified data for in-center dialysis patients prescribed SO as part of routine care. The effectiveness of SO has also been evaluated prospectively by VERIFIE (Velphoro Evaluation of Real-lIfe saFety, effectIveness, and adherencE)—a post-authorization safety study of SO in European dialysis patients.

### Fresenius Kidney Care database studies

#### SO monotherapy in hemodialysis patients switched from other PBs

The effect of switching hemodialysis patients from other PBs to SO monotherapy was evaluated by several retrospective analyses of the Fresenius Kidney Care (FKC) database.

An initial study analyzed data for 1029 adult in-center hemodialysis patients switched to SO monotherapy for up to 6 months [33]. Most patients switched to SO had poor phosphorus control. In total, 424 patients received > 3 consecutive months of SO prescriptions. The proportion of this group achieving in-target serum phosphorus (≤ 5.5 mg/dl) increased approximately two-fold by the end of the 6-month follow-up period (from 15.6% at baseline to 30.4%, *p* < 0.0001), while daily PB pill burden fell from 9.7 to 4.0 pills/day (*p* < 0.0001) [33].

A subsequent longer-term study [34] evaluated 530 in-center hemodialysis patients switched to SO monotherapy, receiving 12 months of continuous prescriptions. Comparisons in PB pill burden, serum phosphorus, and other clinical parameters were made between baseline (the 91-day period prior to SO treatment) and consecutive 91-day intervals of SO treatment (Q1–Q4). PBs received by patients during the baseline period included sevelamer (59.8%), calcium acetate (CaAc) (27.6%), lanthanum carbonate (7.9%), or magnesium carbonate (0.4%), or a switch between these agents (4.3%).

After the switch to SO, mean serum phosphorus progressively decreased (from 6.82 mg/dl at baseline to, respectively, 6.54 mg/dl, 6.37 mg/dl, 6.25 mg/l, and 6.19 mg/dl at Q1, Q2, Q3, and Q4; *p* < 0.0001 vs baseline). The percentage of patients with serum phosphorus ≤ 5.5 mg/dl increased approximately two-fold, rising from 17.7% at baseline to 24.5%, 30.5%, 36.4%, and 36.0% at Q1, Q2, Q3, and Q4, respectively (*p* < 0.0001). Mean daily PB pill burden declined by approximately 50%, from 8.5 pills/day at baseline to between 4.0 and 4.3 pills/day during Q1–Q4 (*p* < 0.0001) [34].

A stratified analysis found consistent reductions in serum phosphorus concentrations and increases in the proportion of patients achieving serum phosphorus ≤ 5.5 mg/dl, irrespective of baseline PB therapy [34]. For patients who switched from sevelamer to SO (*n* = 317), mean serum phosphorus decreased from 6.77 mg/dl at baseline to 6.14 mg/dl by Q4 (*p* < 0.0001). Marked reductions from baseline in serum phosphorus were also observed for patients switching from CaAc to SO (*n* = 146) (from 6.90 mg/dl to 6.31 mg/dl by Q4; *p* < 0.0001), and those switching from lanthanum carbonate to SO (*n* = 42) (from 6.71 mg/dl to 5.93 mg/dl by Q4;* p* < 0.0001). Large reductions (> 50%) in PB pill burden were observed among patients who switched from sevelamer and CaAc. Those switching from sevelamer received 8.9 sevelamer pills/day at baseline vs 4.0–4.4 SO pills/day during Q1–Q4 (*p* < 0.0001). Patients switching from CaAc received 8.9 CaAc pills/day at baseline vs 3.9–4.0 SO pills/day during Q1–Q4 (*p* < 0.0001). Pill burden remained unchanged for patients who switched from lanthanum carbonate (4.4 lanthanum pills/day at baseline vs 4.5–4.7 SO pills/day; *p* = 0.56), although the improvement in phosphorus control with SO paralleled the results for the other PBs.

This study also examined the effectiveness of 1 year of SO monotherapy in the subgroups of Black/African American (*n* = 217) and Hispanic patients (*n* = 87) [34]. Switching to SO among Black/African American patients increased the proportion of those achieving serum phosphorus ≤ 5.5 mg/dl from 14.3% at baseline to 23.0–34.1% during Q1–Q4 (*p* = 0.004), while PB pill burden fell from 8.9 pills/day at baseline to 4.1–4.5 SO pills/day during Q1–Q4 (*p* < 0.0001). Similarly, the proportion of Hispanic patients achieving serum phosphorus ≤ 5.5 mg/dl increased from 18.4% at baseline to between 28.7% and 39.1% during Q1–Q4, and PB pill burden declined from 8.9 pills/day at baseline to 4.1–4.4 SO pills/day (all *p* < 0.0001).

Small reductions in corrected calcium (9.25 mg/dl at baseline vs 9.10 mg/dl at Q4; *p* < 0.0001) and increases in serum intact parathyroid hormone (611 pg/ml at baseline vs 643 pg/ml at Q4; *p* = 0.16) were observed. The extent of the changes in iPTH were similar to those observed in the Phase 3 trial and its extension study [26, 27]. As approximately one third of patients were on calcium-based PBs prior to switching, the withdrawal of calcium loading and progression of hyperparathyroidism may account for these small changes.

The previously described retrospective database analyses used patients’ own data prior to switching to SO, but a concurrent control group was lacking. To address this issue, a retrospective cohort study of hemodialysis patients that utilized a novel design was conducted in order to evaluate the long-term real-world effectiveness of SO, compared with other routinely prescribed PBs [35].

In this study, adult in-center hemodialysis patients maintained on SO therapy (designated “mSO”) for 2 years were compared with an active control group of patients who discontinued SO (“dSO”) within 90 days of their initial prescription and were switched back to other PB(s). All patients had serum phosphorus laboratory values and PB therapy recorded at baseline (3-month period prior to SO initiation) and the final quarter (Q8) of the 2-year follow-up period (Q1–Q8). Key outcomes assessed included achievement of the serum phosphorus target (≤ 5.5 mg/dl) and PB pill burden [35].

**Fig. 1:**
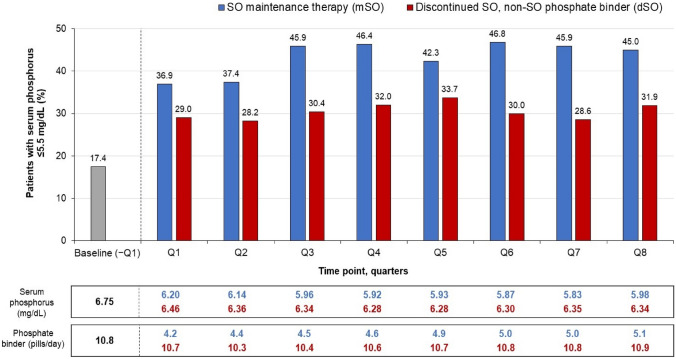
Serum phosphorus control and phosphate binder pill burden among maintenance sucroferric oxyhydroxide (mSO) and discontinued SO (dSO) patients at baseline and during the 2-year follow-up period. Baseline % patients in-range: 20.7% (mSO), 16.1% (dSO); baseline serum phosphorus: 6.61 mg/dl (mSO), 6.8 mg/dl (dSO); baseline phosphate binder pills/day: 8.5 (mSO), 11.6 (dSO)

A total of 818 patients (222 mSO and 596 dSO) were included in the analysis [35]. The proportion of patients achieving serum phosphorus ≤ 5.5 mg/dl during Q1–Q8 increased significantly both in the mSO group [from 20.7% at baseline vs 36.9% at Q1 to 45.0% at Q8 (*p* < 0.001)] and in the dSO group [16.1% at baseline vs 29.0% at Q1 to 31.9% at Q8 (*P* < 0.001)] (Fig. 1). In the mSO group, 45% achieved serum phosphorus ≤ 5.5 mg/dl at Q8 with 3.1 fewer pills/day, compared with baseline (7.5 vs 4.4 pills/day; *p* < 0.001), while 31.9% of dSO patients achieved serum phosphorus ≤ 5.5 mg/dl at Q8 with an unchanged pill burden (9.1 vs 9.3 pills/day; *p* = 0.3). Over 2 years of follow-up, mean serum phosphorus decreased more in the mSO group than in the dSO group (− 0.66 mg/dl vs − 0.45 mg/dl; *p* = 0.014). Mean PB pill burden also decreased in the mSO group (8.5 to 5.1 pills/day; *p* < 0.001), but no change was observed in the dSO group (11.6 to 10.9 pills/day; *p* = 0.2) (Fig. 1). To evaluate the potential bias resulting from SO discontinuation during follow-up, a sensitivity analysis of data for 3047 patients who had received less than 2 years of SO therapy was also performed. These findings confirmed that greater serum phosphorus reductions were achieved with SO than with other PBs. Significant reductions from baseline in serum calcium and small but significant increases in parathyroid hormone were similarly observed in the mSO and dSO groups during the 2-year follow-up period [35].

Overall, this 2-year retrospective comparative database showed patients maintained on SO therapy for 2 years had a greater likelihood of achieving target serum phosphorus concentrations and used 50% fewer PB pills/day, compared with those switched to other routinely prescribed PBs [35].

#### PB-naïve patients prescribed SO monotherapy

A retrospective database study of 172 adult PB-naïve in-center hemodialysis patients prescribed SO therapy for 12 months (Q1–Q4) showed significant increases in the proportion achieving serum phosphorus ≤ 5.5 mg/dl, from 23.7% at baseline to 32.6–38.8% during Q1–Q4 (*p* < 0.0001) [36]. The mean pill burden per quarter ranged from 4.0 to 4.1 SO pills/day. A subgroup analysis of 44 patients who were within their first year of dialysis also found a significant increase in the percentage of patients achieving serum phosphorus ≤ 5.5 mg/dl on SO therapy, from 31.8% at baseline to between 40.9 and 52.4% during Q1–Q4 (*p* < 0.0001).

A longer-term retrospective study assessed a cohort of 59 incident hemodialysis patients in their first year of dialysis who were prescribed SO monotherapy for 2 years (Q1–Q8) [37]. Treatment with SO was associated with a significant reduction in serum phosphorus, from 6.14 mg/dl to 5.49 mg/dl by Q8 (*p* < 0.0001), while the proportion of patients achieving serum phosphorus ≤ 5.5 mg/dl was 37% at baseline, 64% after 1 year (Q4) and 49% after 2 years (Q8) (*p* < 0.001 baseline vs Q1 − Q8 follow-up). Mean SO pill burden ranged from 4.4 to 5.1 SO pills/day.

It should be acknowledged that both of these studies included a relatively small number of patients and have only been published in congress abstract form and not undergone peer review [36, 37].

#### SO therapy in combination with other PBs

The effects of SO prescribed with other PBs were evaluated by a retrospective study of 234 in-center hemodialysis patients [38]. Patients received ≥ 120 days of prescriptions of SO with other PB therapies, including CaAc, lanthanum carbonate and sevelamer, for up to 1 year. For most patients in the cohort (*n* = 196, 84%), SO was added to their baseline PB therapy, while the remainder either received SO along with a different PB(s) to their baseline regimen (*n* = 22, 9%), or were newly initiated on PB combination therapy with SO (*n* = 16, 7%). In the overall study cohort, use of SO in combination with other PBs was associated with significant reductions in serum phosphorus (6.7 mg/dl at baseline vs 6.2–6.3 mg/dl during Q1–Q4; *p* < 0.001) and significant increases in the proportion of patients with serum phosphorus ≤ 5.5 mg/dl (19% baseline vs 33–40% during Q1–Q4; *p* < 0.001). Total PB pill burden initially increased from 12.3 pills/day at baseline to 15.8 pills/day at Q1 (*p* < 0.001) following the addition of SO treatment. However, the number of non-SO PB pills were down-titrated over time, so that by Q4, the mean total PB pill burden was 12.3 pills/day (baseline vs Q4, *p* = 0.9). The mean SO pill burden during the 1-year follow-up period ranged from 4.0 pills/day at Q1 to 4.6 pills/day at Q4. Subgroup analysis of patients who received SO in addition to their baseline PB (*n* = 196) showed that addition of SO to CaAc (*n* = 54) or sevelamer (*n* = 94) was associated with ≥ 2.5-fold increases in the proportion of patients achieving serum phosphorus ≤ 5.5 mg/dl (*p* < 0.001). Overall, this study shows that addition of SO to other PB regimens improves the proportion of patients achieving phosphorus goal, but does not decrease PB pill burden [38].

#### Effect of SO monotherapy on serum albumin and nutritional parameters

Improvements in phosphorus control with conversion to SO may permit or even motivate patients to increase their protein intake, which could be beneficial to the patient despite mitigating the phosphorus reduction by SO. To explore whether this might occur, the impact of SO therapy on serum albumin and other nutritional parameters was evaluated by a retrospective analysis of data for 79 adult in-center hemodialysis patients with hypoalbuminemia (≤ 3.5 g/dl) who were switched to SO for a minimum of 1 year [39]. A matched reference group of patients without hypoalbuminemia at baseline (> 3.5 g/dl; *n* = 79) who were switched to SO was also evaluated [39]. The results showed that both hypoalbuminemic and non-hypoalbuminemic patients switched to SO achieved reductions in serum phosphorus (– 0.40 g/dl and – 0.51 g/dl, respectively) and daily PB pill burden (by 45.7% and 45.1%, respectively). Mean serum albumin concentrations among non-hypoalbuminemic patients remained largely unchanged during the SO follow-up period (4.03 g/dl at baseline vs 3.97–4.01 g/dl during Q1–Q4; *p* = non-significant). In contrast, significant increases in serum albumin concentrations during SO therapy were seen in hypoalbuminemic patients (from 3.41 to 3.50 g/dl during the 6-month baseline period [− Q1 and − Q2] vs 3.69–3.74 g/dl during Q1–Q4; *p* < 0.0001), together with prolonged improvements in other nutritional parameters, including increases in normalized protein catabolic rate, pre- and post-dialysis weight, and serum creatinine [39]. These findings suggest that treatment with SO may enable hemodialysis patients to increase their intake of dietary protein.

#### Effectiveness of SO in peritoneal dialysis patients

A retrospective database analysis evaluated 258 adult US peritoneal dialysis patients prescribed SO monotherapy for up to 6 months [40]. At baseline (3-month period prior to SO prescription), patients’ mean serum phosphorus was 6.59 mg/dl, and 74% had serum phosphorus concentrations > 5.5 mg/dl. During the 6-month SO treatment period, the proportion of patients achieving serum phosphorus ≤ 5.5 mg/dl increased from 26.0% at baseline to 44.4% after 6 months (*p* < 0.001). Mean PB pill burden decreased more than two-fold after patients switched to SO, from 10 PB pills/day at baseline to 4.3 SO pills/day during the follow-up period (*p* < 0.0001).

### DaVita Inc. database study

If a reduced PB pill burden increases adherence, patients might request prescription refills more regularly. To assess for this effect, an analysis was performed using electronic health records from the DaVita Inc. database and pharmacy service to evaluate PB pill burden, adherence, and serum phosphorus control in 490 prevalent in-center hemodialysis adults who had switched to SO therapy from other PB(s) as part of routine care [41]. The 6 months prior to SO prescription was designated as baseline and the 6-month period after the first SO prescription as follow-up.

As observed in studies of the FKC hemodialysis patient population, switching to SO therapy was associated with a significant reduction from baseline in mean PB pill burden from 10.8 pills/day to 5.5 pills/day (*p* < 0.001), and increases in the proportion of patients achieving serum phosphorus ≤ 5.5 mg/dl from 22.0 to 30.0% (*p* < 0.001) [41].

A novel feature of this study was its evaluation of whether switching to SO from other PB(s) was associated with improved adherence to therapy, based on changes in medical possession ratio (MPR) for 30 patients who were not enrolled in the automated prescription refill service [41]. In these patients, mean total PB MPR increased significantly over time, from 0.68 at baseline to 0.80 during SO follow-up (*p* = 0.01), supporting that switching to SO was associated with improved treatment adherence. Improvements in MPR suggest better adherence to SO may be related to lower pill burden and drive the observed improvements in phosphorus.

### Fresenius Medical Care EuCliD® database study

A retrospective study evaluated the real-world effectiveness of SO in European hemodialysis patients, utilizing data from the EuCliD^®^ database to analyze a cohort of hemodialysis patients from five European countries who were prescribed SO for up to 1 year [42]. Serum phosphorus and PB pill burden of the overall cohort (*n* = 1,096) were compared between a 3-month baseline period prior to SO initiation and four quarterly periods of SO therapy (Q1 − Q4). In addition, three patient subgroups were separately analyzed: (1) PB-naïve patients treated with SO monotherapy (SO; *n* = 188); (2) PB-pretreated patients switched to SO monotherapy (PB → SO; *n* = 53); and (3) PB-pretreated patients receiving SO added to another PB (PB + SO; *n* = 796).

In the overall cohort, serum phosphorus decreased significantly from 5.83 mg/dl at baseline to 5.48–5.24 mg/dl during Q1–Q4 (*p* < 0.0001 vs baseline) [42]. The proportion of patients with serum phosphorus ≤ 5.5 mg/dl increased from 41.3% at baseline to between 56.2 and 62.7% during Q1–Q4 (*p* < 0.0001 vs baseline). The total PB pill burden decreased from 6.3 pills/day at baseline to between 5.0 and 5.3 pills/day over the 1-year SO treatment period.

In the subgroup analysis, the proportion of patients achieving serum phosphorus ≤ 5.5 mg/dl increased to the greatest extent in the SO subgroup (from 49.5% at baseline to 62.5–75.2% during Q1–Q4; *p* < 0.0001 for Q1, Q2, Q3) and in the PB + SO subgroup (from 38.1% at baseline to 53.9–60.9% during Q1–Q4; *p* < 0.0001 for all quarters) [42]. There were no statistically significant changes in the proportion of patients achieving serum phosphorus ≤ 5.5 mg/dl in the PB → SO group. In the PB + SO group, the total mean number of PB pills prescribed during SO follow-up remained unchanged (6.5 pills/day at baseline vs 6.0 − 6.2 pills/day during Q1–Q4), while daily PB pill burden marginally increased for PB → SO patients, from 2.1 pills/day at baseline to between 2.6 and 2.8 pills/day. The mean SO pill burden was relatively low across all subgroups analyzed (2.1 − 2.8 pills/day).

Overall, the EuCliD^®^ database analysis indicated that administration of SO either as monotherapy to PB-naïve patients or as add-on therapy to an existing PB therapy regimen was associated with improvements in serum phosphorus control and a low daily pill burden [42].

### VERIFIE post-authorization safety study

The long-term effectiveness of SO for serum phosphorus control was prospectively evaluated by the VERIFIE post-authorization safety study [43]. VERIFIE enrolled 1406 adult hemodialysis or peritoneal dialysis patients from seven European countries, all of whom had been prescribed SO in accordance with the product label. The planned follow-up period for each patient was 12–36 months.

**Fig. 2:**
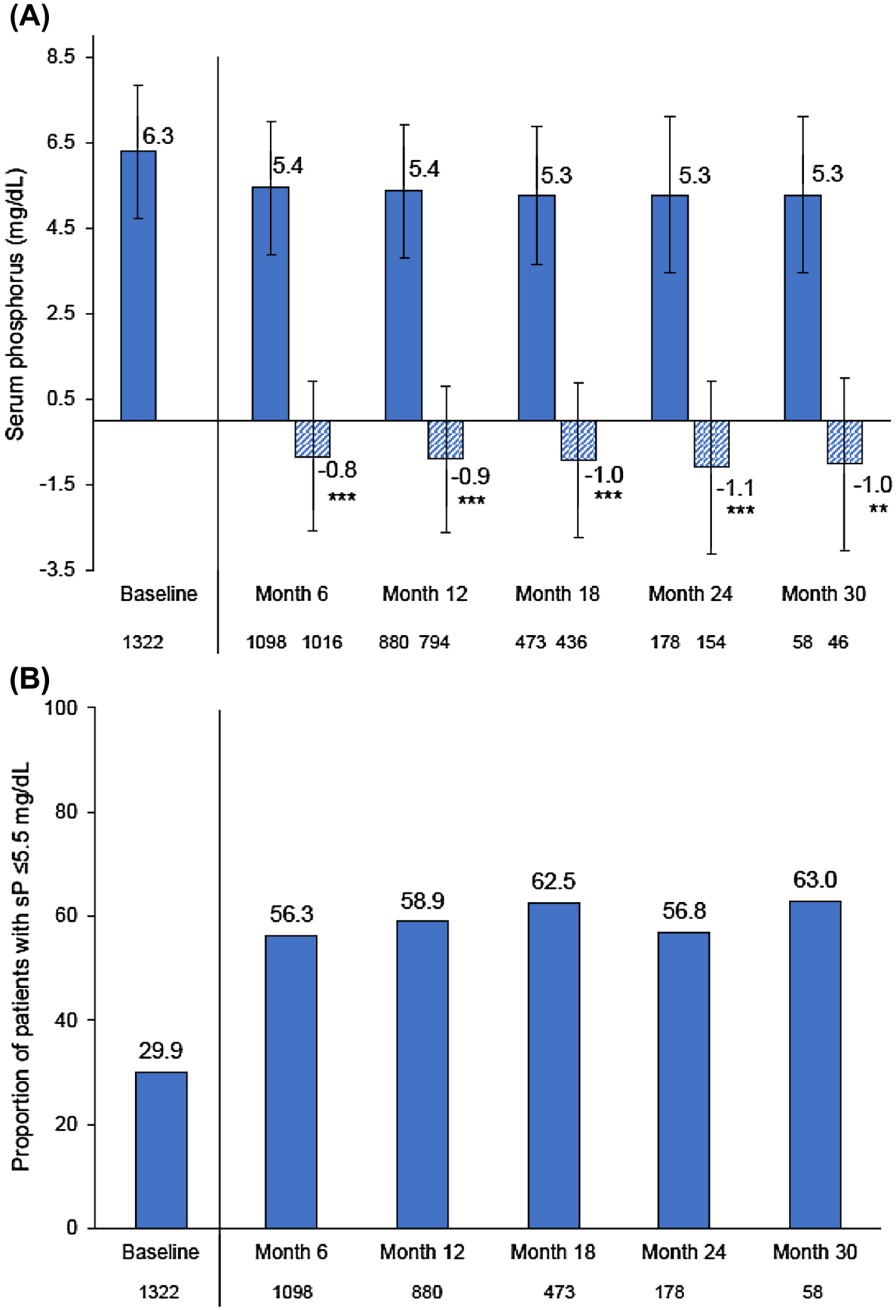
VERIFIE study: serum phosphorus control during the observation period (full analysis set; *N* = 1322). **A** Mean ± SD phosphorus concentrations and changes from baseline over time. **B** Proportion of patients with serum phosphorus ≤ 5.5 mg/dl. **p < 0.01, ***p < 0.001 vs baseline. On panel **A**, bars show mean values and whiskers represent standard deviations. *SD*, standard deviation; *sP*, serum phosphorus

In total, 1322 patients were included in the effectiveness analysis: the majority (*n* = 1169; 88.4%) were receiving hemodialysis and 153 (11.6%) undergoing peritoneal dialysis [43]. SO therapy was associated with significant reductions from baseline in mean serum phosphorus (6.3 mg/dl vs 5.3 mg/dl at Month 30; Δ baseline: − 1.0 mg/dl; *p* < 0.01) (Fig. 2a). The proportion of patients achieving serum phosphorus ≤ 5.5 mg/dl increased from 30% at baseline to between 47 and 63% during follow-up (Fig. 2b). The mean daily SO dose during the overall observation period was 2.3 SO pills/day. Forty-five percent of patients in VERIFIE were prescribed SO in combination with other PBs. A stratified analysis showed similar serum phosphorus reductions among patients who received SO in combination with other PBs and those who received SO monotherapy. The mean daily SO pill burden during the observation period was also similar between the SO monotherapy and SO combination groups (2.5 pills/day and 2.3 pills/day, respectively) [43].

### Effect of SO therapy on hospitalizations and potential cost-savings

Treatment with SO may be associated with a reduced rate of hospitalizations, compared with other PB therapies.

An observational study analyzed data from 24 end-stage renal disease seamless care organizations in the USA to assess hospital admission rates of dialysis patients prescribed different PB therapies over a 3-year period (2016–2018) [44]. The hospitalization rate (per 100-member months [MM]) was lower among patients treated with SO (7.97 per 100-MM) compared with those treated with sevelamer (10.52 per 100-MM), CaAc (11.28 per 100-MM), ferric citrate (9.54 per 100-MM), or lanthanum carbonate (8.86 per 100-MM) [44].

The effect of SO monotherapy vs other routinely prescribed PBs on the incidence of hospital admissions in US hemodialysis patients was evaluated by the 2-year retrospective comparative cohort study by Coyne and colleagues [35]. Patients maintained on SO therapy for 2 years had 35.6 fewer hospitalizations per 100 patient-years than patients who discontinued SO within 90 days and switched to other PBs [incidence rate ratio = 0.75 (95% confidence interval 0.58–0.96)]. Using in-patient expenditure data for hemodialysis patients from the 2018 US Renal Data System Annual Data Report, use of SO instead of other PBs was associated with a potential annual cost saving of $566,295 per 100 patients [35].

A subsequent economic analysis applied the hospitalization data from the study [35] to potential hospitalization cost-savings with SO vs other PBs in five European countries [45]. The results showed that treatment with SO is likely to result in inpatient cost-savings of €118,922, €451,714, €227,940, €125,750, and €314,282 per 100 patient-years lived in, respectively, France, Germany, Italy, Spain, and the UK [45].

## Safety and tolerability data for SO in real-world settings

### VERIFIE post-authorization safety study

Primary safety endpoints of VERIFIE included the incidence of adverse drug reactions (ADRs) and medical events of special interest (MESIs) (defined as adverse events of GI bleeding, diarrhea, and iron accumulation irrespective of their relationship to SO) [43]. Physicians’ evaluations of the potential masking effect of the stool discoloration due to SO treatment on GI bleeding diagnosis, iron-related parameters (ferritin, TSAT, and hemoglobin), and fatal events were also assessed.

In total, 1,365 patients were included in the safety analysis set. The mean observation period was 420 days, and 59% of patients were treated with SO for 12 months or longer. A total of 531 patients (39%) in the safety analysis set had ≥ 1 ADR during treatment with SO (Table 2). The most common ADRs were GI disorders, mainly diarrhea and discolored feces, reported by 194 (14%) and 128 (9%) patients, respectively. Serious ADRs were reported for 26 patients (2%). Overall, 250 patients (18%) had ≥ 1 MESI during the study, the most frequent of which were GI disorders [43].

**Table 2:** Adverse drug reactions occurring in ≥ 1.0% of patients by system organ class and preferred term in the VERIFIE PASS study

System Organ Class	Safety Analysis Set (*N* = 1,365)
Preferred Term	Patients, *n* (%)
Patients with at least 1 ADR	531 (38.9)
Gastrointestinal disorders	436 (31.9)
Diarrhea	194 (14.2)
Discolored feces	128 (9.4)
Abnormal feces	48 (3.5)
Constipation	40 (2.9)
Abdominal pain	38 (2.8)
Nausea	36 (2.6)
Soft feces	20 (1.5)
Vomiting	17 (1.2)
Dyspepsia	16 (1.2)
Injury, poisoning, and procedural complications	59 (4.3)
Off-label use	29 (2.1)
General disorders and administration-site conditions	56 (4.1)
Drug ineffective	26 (1.9)
Treatment noncompliance	15 (1.1)
Product issues	24 (1.8)
Product taste abnormal	23 (1.7)

All ADRs were coded based on MedDRA Version 22.0 terminology into System Organ Class and Preferred Terms

*ADR*, adverse drug reaction; *MedDRA*, Medical Dictionary for Regulatory Activities; *PASS*, post-authorization safety study

A total of 217 patients (16%) reported diarrhea. It tended to occur soon after SO initiation and was generally mild (53%) or moderate (40%) in severity. In most patients, the first event of diarrhea resolved within 2 weeks of initial onset. These findings were consistent with those previously reported in the Phase 3 study, in which diarrhea was mainly mild and transient in nature [26, 27].

GI bleeding occurred in 38 patients while being treated with SO during the study (46 events). Most patients (*n* = 32, 84%) had risk factors for GI bleeding, including medication (use of anticoagulant therapy), history of GI bleeding, or medical conditions and/or disease with increased bleeding risk [43]. No clinically significant delays in GI bleeding diagnosis owing to SO-related stool discoloration were reported during the study.

A total of 119 fatal events (8.7%) occurred during the study, none of which were considered related to SO treatment. Overall, the VERIFIE study confirmed that SO has a good safety and tolerability profile [43], consistent with observations in the Phase 3 trial and its extension study [26, 27].

### Impact of SO therapy on iron parameters and anti-anemia medication use

The effect of SO therapy on iron parameters and anti-anemia medication use in the real-world setting has been evaluated by several studies. In the VERIFIE study, SO therapy was associated with small increases in mean serum ferritin (from 377 µg/l at baseline up to 444 µg/L at Month 24; Δ baseline: + 75 µg/l; *p* < 0.05) and TSAT (from 26.1% at baseline up to 29.0% at Month 3; Δ baseline: + 2.1%; *p* < 0.001). A subgroup analysis stratifying patients by concomitant IV/oral iron use (yes vs no) indicated that increases in these iron parameters were mainly driven by iron therapy use, as ferritin values did not increase in the latter subgroup [43]. MESIs of iron overload were reported for two patients during the study. However, both were receiving concomitant IV iron therapy, and one of them had an iron utilization disorder [43].

Retrospective observational studies of both hemodialysis and peritoneal patients have also reported small but significant initial increases in ferritin and TSAT during treatment with SO [33–36, 41–43]. The findings are consistent with the minimal iron absorption from SO reported in the Phase 3 trial and its extension [30].

Use of SO may result in a small reduction in overall IV iron use. A decline in the usage and/or dose of IV iron or ESA among patients treated with SO has been reported by real-world studies in the USA and Europe [33–35, 42, 43]. These findings are consistent with the results of the Phase 3 study and its extension, in which SO treatment was accompanied with a small reduction in the proportion of patients receiving concomitant IV iron and ESA use over 1 year [30].

## Limitations

This review has some limitations. First, it was a narrative rather than systematic literature review, and therefore may not have captured all published studies evaluating the use of SO in real-word clinical practice. Second, several of the SO studies described were retrospective in nature and used data from electronic medical records, which were not specifically collected for the purposes of clinical research. Consequently, some variables of interest were not captured, including data relating to SO tolerability, treatment adherence, reasons for treatment initiation, and information relating to patients’ nutritional habits/dietary phosphate intake. Finally, it should be acknowledged that the authors of this review also participated as investigators in some of the SO studies described in the text.

## Conclusions

Evidence from prospective randomized controlled trials and real-world observational studies has demonstrated that SO is an effective and well-tolerated PB therapy for control of serum phosphorus concentrations in dialysis patients and is associated with a low daily pill burden.

The effectiveness of SO therapy in the real-world clinical setting has been extensively evaluated by a range of observational studies conducted in a large number of hemodialysis (> 6400) and peritoneal dialysis (~ 500) patients in the USA and Europe in both retrospective and prospective cohorts. In line with the Phase 3 clinical trial program data, these studies have demonstrated that SO can effectively reduce serum phosphorus with a lower daily pill burden than many other commonly prescribed PBs, which may translate into better treatment adherence. The studies have also shown that SO is effective when prescribed in a variety of treatment settings, including as monotherapy among patients switching from other PBs [33–35], as first-line therapy in PB-naïve patients [36, 37], or when used in combination with other PB therapies [38, 43].

It is important to note that the patients included in these real-world studies were a selected group prescribed SO as part of routine clinical practice and therefore may not be representative of the overall dialysis patient population with respect to the severity of their hyperphosphatemia. In the US patient cohorts, initiation of SO therapy significantly improved serum phosphorus control, but many patients did not achieve target phosphorus concentrations (≤ 5.5 mg/dl). This observation illustrates the challenge of managing hyperphosphatemia in the real-world setting and indicates the need for a combined treatment approach. The use of SO as part of an individualized, multipronged intervention in conjunction with nutritional counseling, dialysis optimization, and the judicious use of vitamin D therapy, may further help improve serum phosphorus control in patients with hyperphosphatemia. The safety profile of SO therapy, as demonstrated in the clinical trials, was confirmed in the real-world setting by the prospective VERIFIE post-authorization safety study, which included patients from > 1,300 patients undergoing hemodialysis or peritoneal dialysis [43].

In summary, SO offers an effective and well-tolerated treatment option for the control of serum phosphorus concentrations among dialysis patients with hyperphosphatemia in the real-world clinical setting.

## Data Availability

Not applicable.
